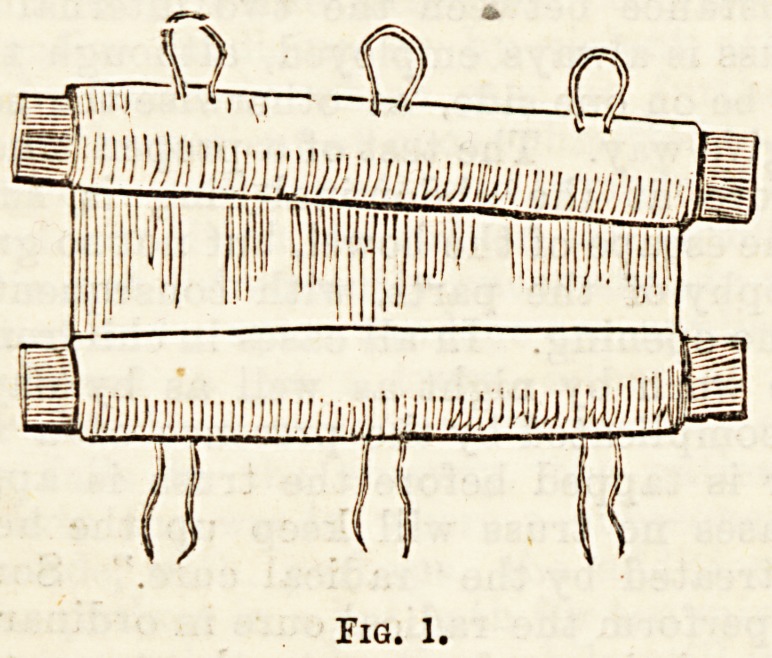# Treatment of Pott's Fracture

**Published:** 1893-02-04

**Authors:** 


### The Hospital Clinic.

[*The Editor will be glad to receive offers of co-operation and contributions from members of the profession. All letters should be addressed to* The Editor, The Lodge, Porchester Square, London, W.]

### ROYAL INFIRMARY, EDINBURGH.

### Treatment of Pott's Fracture.

The fracture of the fibula usually associated with the name of Percival Pott is one of the commonest met with in surgical practice. It is usually the result of a sudden sharp twist such as occurs when the foot slips from the kerbstone, or when one jumping from a height lands on the inner side of the foot. Under such conditions the weight of the body is transmitted, through the astragalus, to the external malleolus, pushing it forcibly outwards, and so bending the fibula, which, being thin and weak for a short distance above its articulation with the tibia, readily snaps across.

The exact seat of the fracture described by Pott is about three inches above the tip of the external malleolus, or, in other words, about the apex of the subcutaneous triangle at the lower end of that bone. Not only is the fibula broken in these cases, but, in addition, the internal lateral ligament of the ankle joint is very frequently torn. Some writers believe that the tip of the inner malleolus breaks oftener than the ligament tears, so that in a majority of cases the condition is really one of fracture of both bones.

There are a certain number of cases in which the

Fig. 1.

injury takes place by a twisting inwards of the foot, whereby the inner malleolus sustains more injury than its fellow.

The displacements resulting vary according to the manner in which the accident occurs. In the commoner injury, in which the foot is twisted out, the deformity is threefold, the most evident alteration being a marked eversion of the foot, produced by the force causing the lesion, and kept up by the peroneal muscles which pass behind the outer ankle. Should the internal malleolus be chipped off, the weight of the heel carries it backwards, its support being gone, at the same time as the toes are pointed by the unrestrained action of the tendo Achillis.

The diagnosis of the condition is made by observing the displacements just named, and considering them in association with the history of the injury. Pain and crepitus will be elicited by pressure over the broken bone as well as at some distance from it, and the patient will be quite unable to bear his weight on the affected limb.

Almost all cases of Pott's fracture can be treated by the ordinary Box Splint, an appliance which, in addition to its efficacy has the great advantage that it can be constructed from materials obtainable anywhere.

Having diagnosed this fracture, all the appliances for its treatment should be got ready before the "setting" is commenced. These are two pieces of wood about a quarter of an inch thick, long enough to extend from the bend of the knee to a short distance beyond the sole of the foot, and a little wider than the limb. In an emergency a few layers of strong pasteboard would serve the purpose. These splints are rolled into the opposite ends of a sheet or bath towel, so that together they make three sides of a box (Fig. 1). In folding the splints into the sheets it should be borne in mind that the limb is a little broader above than below, and allowance made for this difference by folding one side somewhat obliquely. The difficulty in getting the box to the desired size will be less if only one end of the sheet be unrolled. The lid of the box is formed by two hand-towels folded to the width of the front of the leg and about half its length. The other requisites are three pieces of bandage about two yards long for slip knots, a domette bandage, and a quantity of absorbent wool for padding. When wool cannot be had, a good substitute is obtained by pulling down any old knitted garment. The appliances should all be "fitted" on the sound leg so as to avoid interfering with the damaged one as much as possible.

Everything being ready, the fracture has now to be set. An assistant seizes the foot with one hand behind the heel and the other grasping the toes, and gently draws upon it in such a way as to counteract the three prominent displacements, while the surgeon moulds the bones into position. When the bones are accurately set the ball of the great toe, the internal malleolus, and the inner edge of the patella should be in the same straight line. Having done so, the leg is laid into the splint, all bony prominences, especially the condyles and malleoli, are protected with wool, and special pads are inserted where they will help to retain the bones in position. The sides are folded up, the two towels laid in front to form the lid, and the whole fixed by three slip knots, of which the middle one should be tightened first to avoid displacing the ends. The foot is kept at right angles with the leg, and, if necessary, inverted by a figure of eight bandage.

The leg so encased may lie on the bed, protected from pressure by a cage, or it may be swung in a Salter's cradle, in which case the posterior aspect or floor of the box should be strengthened by a piece of wood similar to the sides.

The parts are examined every day at first lest any displacement should take place. It is to facilitate this that *two* towels are used to form the lid. The middle slip knot is undone, the towels folded up and down, and the limb at once exposed without interfering with the splint.

At the end of three or four weeks, the swelling and all pain having gone, a plaster of paris case may be applied, and the patient allowed to go about with crutches for a week or two, after which massage, exercise, and careful use will restore the part to its former condition.

In cases where the eversion is more than usually well marked the internal splint of Dupuytren may be used, or when the falling back of the heel predominates it may be counteracted by the stirrup or horse-shoe splint, but, as a rule, these are unnecessary.

## Figures and Tables

**Fig. 1. f1:**